# Rural residents' willingness to participate in basic medical insurance and influential factors: A survey of three provinces in China

**DOI:** 10.3389/fpubh.2022.1037763

**Published:** 2022-11-10

**Authors:** Guomei Tian, Jinpeng Xu, Ting Zhang, Hongyu Zhang, Jian Liu, Qi Shi, Fangmin Deng, Fangting Chen, Jingran He, Qunhong Wu, Zheng Kang, Hui Wang

**Affiliations:** ^1^Department of Nuclear Medicine, The Fourth Hospital of Harbin Medical University, Harbin, China; ^2^School of Health Management, Harbin Medical University, Harbin, China; ^3^Dushu Lake Hospital Affiliated to Soochow University, Suzhou, China

**Keywords:** basic medical insurance, willingness, influential factors, rural residents, China

## Abstract

**Background:**

Rural residents' participation in medical insurance has a significant relationship to the affordability of their medical care. This study aims to investigate the willingness of rural residents to participate in basic medical insurance for urban and rural residents and its determinants so as to enhance their willingness to participate in medical insurance.

**Methods:**

Data were obtained from 1,077 validated questionnaires from rural residents. Chi-square test and multiple logistic regression analysis were adopted to analyze determinants of rural residents' willingness to participate in basic medical insurance for urban and rural residents.

**Results:**

94.3% of respondents were willing to participate in basic medical insurance for urban and rural residents and this was associated with the familiarity with the medical insurance policies [OR = 2.136, 95% CI (1.143, 3.989)], the reasonability of medical insurance premiums [OR = 2.326, 95% CI (0.998, 5.418)], the normality of doctors' treatment behavior [OR = 3.245, 95% CI (1.339, 7.867)] and the medical insurance's effectiveness in reducing the economic burden of disease [OR = 5.630, 95% CI (2.861, 11.079)].

**Conclusion:**

Even though most respondents were willing to participate in basic medical insurance for urban and rural residents, some aspects need to be improved. The focus should be on promoting and regulating the behavior of medical staff. Financing policies and reimbursement of treatment costs need to be more scientifically developed. A comprehensive basic healthcare system needs to be optimized around the core function of “hedging financial risks”.

## Introduction

Since 1998, basic medical insurance had been proposed and established in China. The basic medical insurance divided the insured population into employees and residents. Residents are classified as rural or urban according to their status, with the new rural cooperative medical insurance and the basic medical insurance for urban residents catering to the needs of these two groups, respectively. As the economy develops, basic medical insurance continues to make progress in addressing medical needs and improving the health of the population. In particular, the health status of rural insured residents has been greatly improved as a result (1).

However, the problems of scattered medical insurance institutions, large disparities between urban and rural medical coverage, and inequitable allocation of health resources still exist (2–4). Faced with this situation, in 2016, the Chinese government called for the integration of the basic medical insurance for urban residents and the new rural cooperative medical insurance system into a new system called Basic Medical Insurance for Urban and Rural Residents (BMIURR). This new BMIURR covers all urban and rural residents, except those insured under the urban employee basic medical insurance. This means that urban and rural residents are no longer restricted by geography and status and can enjoy uniform medical insurance payments and participation. After the integration of basic medical insurance, rural residents have equitable access to better medical services and greater protection (5, 6).

The occurrence of any behavior may be driven by willingness (7). Willingness has a certain degree of spontaneity, and it can accurately predict actual behavior when not disturbed by situational factors (8). As a public policy in which residents voluntarily participate, their willingness to participate and support the policy is directly related to the coverage and sustainability of the medical insurance system. Even with the large number of rural residents moving to the cities and towns, the population of rural residents still accounts for about 35% of China's total population (9). To promote the development of urban-rural integration and reduce rural poverty due to illness, promoting active participation in BMIURR by rural residents is the primary prerequisite to ensure the sustainability of this basic medical insurance.

In examining Malaysian residents' willingness to enroll in the newly introduced community health insurance, Shafie and Hassali (10) found that culture, monthly household income, and the presence of chronic diseases had a significant effect on their willingness to enroll. Duku et al. (11) found that because of adverse selection, people who have used health services or are expected to use them are more likely to participate in health insurance, while most people who have not used health insurance will be reluctant to participate. From the perspective of rural residents' participation behavior, Haile et al. (12) found that 78% of the respondents were willing to join the newly introduced community health insurance scheme in 2010, where socioeconomic and social capital factors had a significant effect on the willingness to participate. Indians who are not well-educated are not very conscious of health precautions, so they are not very willing to take insurance (13).

To the best of our knowledge, few studies have examined the willingness to participate in health insurance among rural groups in China. Therefore, the purpose of this study was to examine the willingness of rural residents to participate in medical insurance. Three representative cities in eastern, western, and northeastern China, namely Shandong, Shanxi, and Liaoning, were selected as study sites to investigate rural residents in these areas and their willingness to participate in BMIURR and to determine the most influential factors regarding their participation. The results can provide information and suggestions for health policy-makers and administrators of medical and health institutions, refine the medical insurance policy, further promote the development of BMIURR, and effectively protect the lives and economies of rural residents.

## Methods

### Data collection

A cross-sectional questionnaire survey was used to analyze the determinants of rural residents' willingness to participate in the basic medical insurance for urban and rural residents. A face-to-face questionnaire survey was conducted in Shandong, Shanxi, and Liaoning provinces from January to December 2019 using a random sampling method. The questionnaire was designed to survey respondents on a range of information, including demographic status, perceptions of medical insurance, and willingness to participate. The results of questionnaires from people living in rural areas were selected as the sample data for this study. We followed the formula below in calculating the sample size for this sampling.


$$\begin{array}{l} n\;=\;\frac{z^{2}\;*\;P\;*\;\left(1\;-\;P\right)}{d^{2}}\; \end{array}$$

where *z* determines the confidence level, and the *z* value is generally 1.96 corresponding to the 95% confidence level we chose. *P* is the percentage of a feature in the target population, which is generally set to 0.5 if no prior data are available. *d* is the acceptable precision/accuracy level, generally, we took 0.03. Therefore, according to the formula, 1,067 samples should be investigated in this study. Because of the possible variation in questionnaire return rates, we ended up surveying a sample of 1,096. After quality screening to exclude the 19 unqualified samples, 1,077 valid questionnaires were finally retained, with a valid return rate of 98.27%.

### Measurement and variables

The dependent variable of the study was the willingness of rural residents to participate in health insurance. Respondents were asked to indicate whether they would like to participate in the BMIURR (1 = No, 2 = Yes).

Independent variables included in the modeling were demographic characteristics (age, gender, marital status, and education), socio-economic status (annual household income level, living arrangement, and low-income family), and physical condition (chronic disease). In addition, based on Donabedian's “Structure-Process-Outcome” quality model (14), their perception of medical insurance was captured on the evaluation index constructed in the framework of the “system design-operation process-operation effect” (the familiarity with the medical insurance policies, the reasonability of medical insurance premiums, the satisfaction with medical insurance compensation coverage, the convenience with the medical insurance compensation process, the normality of doctors' treatment behavior, and the effectiveness of medical insurance in reducing the economic burden of disease). 5-point Likert scale were used to measure respondents' perceptions of the reasonability of medical insurance premiums, the convenience with the medical insurance compensation process, and the normality of doctors' treatment behavior. The normality of doctors' treatment behavior was based on residents' responses to the question, “Do you think physicians' practice is normality?” (ranging from 1, very normative, to 5, very non-normative).

### Statistical analysis

Data were analyzed using SPSS 22.0 (IBM Corp. Released 2013. IBM SPSS Statistics for Windows, Version 22.0. Armonk, NY: IBM Corp.). Descriptive statistical analysis was used to describe the characteristics of the respondents and their willingness to participate in BMIURR in general. The chi-square test was used to determine the significance of the association between willingness to participate and independent variables of different categories and levels. Multiple logistic regression was constructed to identify factors influencing willingness to participate in BMIURR after adjusting for the effects of other independent variables. OR, *P*-values and 95% CI were calculated, and two-sided statistical significance was set at *P* < 0.05.

## Results

### Characteristics of participants

A total of 1,077 respondents were included in the cross-sectional analysis, of which 47.1% were male and 52.9% were female. The age was mainly concentrated between 30 and 44 years old. The percentage of respondents whose education level is “junior high school” was 47.6%. The majority of respondents were married (79.5%) and the type of residence was mainly two-generation living (64.4%). The majority of household incomes were above 50,000 RMB, accounting for 36.5% of the total. In terms of health status, 90.0% of the respondents suffered from chronic diseases in the past 6 months (Table 1).

**Table 1:** Demographic characteristics of the participants (*n* = 1,077).

**Characteristics**	***N* (%)**	**Willingness to participate in the basic medical insurance for urban and rural residents**		**χ^2^**	***P*-value**
		**No (%)**	**Yes (%)**		
Observations	1,077	61 (5.7)	1,016 (94.3)		
**Age**				11.261	0.010
≤29	196 (18.2)	18 (9.2)	178 (90.8)		
30–44	513 (47.6)	33 (6.4)	480 (93.6)		
45–59	252 (23.4)	6 (2.4)	246 (97.6)		
≥60	116 (10.8)	4 (3.4)	112 (96.6)		
**Gender**				0.752	0.386
Male	507 (47.1)	32 (6.3)	475 (93.7)		
Female	570 (52.9)	29 (5.1)	541 (94.9)		
**Marital status**				16.954	0.001
Unmarried	126 (11.7)	17 (13.5)	109 (86.5)		
Married	856 (79.5)	41 (4.8)	815 (95.2)		
Divorce	49 (4.5)	2 (4.1)	47 (95.9)		
Lost spouse	46 (4.3)	1 (2.2)	45 (97.8)		
**Education**				4.797	0.091
Primary school and below	199 (18.5)	5 (2.5)	194 (97.5)		
Junior High School	513 (47.6)	31 (6.0)	482 (94.0)		
High school and above	365 (33.9)	25 (6.8)	340 (93.2)		
**Annual household income level**				1.771	0.413
<30,000 RMB	383 (35.6)	26 (6.8)	357 (93.2)		
30,000–50,000 RMB	301 (27.9)	17 (5.6)	284 (94.4)		
>50,000 RMB	393 (36.5)	18 (4.6)	375 (95.4)		
**Living arrangement**				28.191	<0.001
Live alone	117 (10.9)	18 (15.4)	99 (84.6)		
Live with contemporaries	167 (15.5)	5 (3.0)	162 (97.0)		
Live with two generations	694 (64.4)	30 (4.3)	664 (95.7)		
Live with three generations	55 (5.1)	6 (10.9)	49 (89.1)		
Live with isolated generations	44 (4.1)	2 (4.5)	42 (95.5)		
**Low-income family**				0.048	0.827
Yes	47 (4.4)	3 (6.4)	44 (93.6)		
No	1,030 (95.6)	58 (5.6)	972 (94.4)		
**Chronic disease**				1.871	0.171
Yes	108 (10.0)	3 (2.8)	105 (97.2)		
No	969 (90.0)	58 (6.0)	911 (94.0)		
**The familiarity with the medical insurance policies**				20.590	<0.001
Familiar	666 (61.8)	21 (3.2)	645 (96.8)		
Unfamiliar	411 (38.2)	40 (9.7)	371 (90.3)		
**The reasonability of medical insurance premiums**				31.222	<0.001
Very reasonable	313 (29.1)	11 (3.5)	302 (96.5)		
Reasonable	208 (19.3)	10 (4.8)	198 (95.2)		
General	242 (22.5)	12 (5.0)	230 (95.0)		
Unreasonable	152 (14.1)	4 (2.6)	148 (97.4)		
Very unreasonable	162 (15.0)	24 (14.8)	138 (85.2)		
**The satisfaction with medical insurance compensation coverage**				2.174	0.140
Satisfied	399 (37.0)	28 (7.0)	371 (93.0)		
Dissatisfied	678 (63.0)	33 (4.9)	645 (95.1)		
**The convenience with the medical insurance compensation process**				15.977	0.003
Very convenient	63 (5.8)	4 (6.3)	59 (93.7)		
Convenient	163 (15.2)	10 (6.1)	153 (93.9)		
General	307 (28.5)	6 (2.0)	301 (98.0)		
Inconvenient	287 (26.6)	16 (5.6)	271 (94.4)		
Very inconvenient	257 (23.9)	25 (9.7)	232 (90.3)		
**The normality of doctors' treatment behavior**				27.192	<0.001
Very normative	119 (11.0)	5 (4.2)	114 (95.8)		
Normative	183 (17.0)	11 (6.0)	172 (94.0)		
General	467 (43.4)	18 (3.9)	449 (96.1)		
Non-normative	216 (20.1)	11 (5.1)	205 (94.9)		
Very non-normative	92 (8.5)	16 (5.7)	76 (82.6)		
**The effectiveness of medical insurance in reducing the economic burden of disease**				69.970	<0.001
Effective	837 (77.7)	21(2.5)	816 (97.5)		
Not effective	240 (22.3)	40 (16.7)	200 (83.3)		

### Rural residents' willingness to participate in BMIURR

This study shows that 94.3% of the respondents were willing to participate in the basic medical insurance for urban and rural residents. Age, marital status, living arrangement, the familiarity with the medical insurance policy, the reasonability of medical insurance premiums, the convenience with the medical insurance compensation process, the normality of doctors' treatment behavior, the effectiveness of medical insurance in reducing the economic burden of disease factors were significantly associated with the respondents' willingness to participate in BMIURR (Table 1).

### Results of multiple logistic regression

To further explore the factors influencing the public's willingness to participate in BMIURR, we included the statistically significant variables in the univariate analysis in a multiple logistic regression analysis. Multiple logistic regression results showed that the deeper the knowledge of health insurance [OR = 2.136, 95% CI (1.143, 3.989)], the stronger the willingness to participate in basic health insurance for urban and rural residents. The public's willingness to participate in health insurance is higher among those who believe health insurance costs are generally reasonable compared to those who believe health insurance costs are unreasonable [OR = 2.326, 95% CI (0.998, 5.418)] (*P* = 0.05, hint of significance). The public's willingness to participate in insurance is stronger for those who perceive doctors' treatment behavior as generally regulated than for those who perceive them as very irregular [OR = 3.245, 95% CI (1.339, 7.867)]. The public that believes health insurance reduces the financial burden of illness is more likely to participate than those who believe that it does not [OR = 5.630, 95% CI (2.861, 11.079)] (Table 2).

**Table 2:** Logistic regression results of willingness to participate in urban and rural residents' basic medical insurance (*n* = 1,077).

**Characteristics**	**OR**	**95%CI**	***P*-value**
**Age**			0.135
≤29	0.631	(0.096, 4.138)	0.631
30–44	0.295	(0.070, 1.254)	0.098
45–59	0.837	(0.186, 3.760)	0.817
≥60 (reference)			
**Marital status**			0.407
Unmarried	0.174	(0.013, 2.375)	0.190
Married	0.366	(0.033, 4.106)	0.415
Divorce	0.913	(0.056, 14.812)	0.949
Lost spouse (reference)			
**Living arrangement**			0.011
Live alone	0.445	(0.057, 3.503)	0.442
Live with contemporaries	1.550	(0.229, 10.484)	0.653
Live with two generations	2.627	(0.436, 15.834)	0.292
Live with three generations	0.872	(0.132, 5.742)	0.886
Live with isolated generations (reference)			
**The familiarity with the medical insurance policies**			
Familiar	2.136	(1.143, 3.989)	0.017
Unfamiliar (reference)			
**The reasonability of medical insurance premiums**			0.109
Very reasonable	1.771	(0.714, 4.391)	0.218
Reasonable	1.546	(0.600, 3.985)	0.367
General	2.326	(0.998, 5.418)	0.050
Unreasonable	4.039	(1.267, 12.878)	0.018
Very unreasonable (reference)			
**The convenience with the medical insurance compensation process**			0.223
Very convenient	0.761	(0.200, 2.895)	0.688
Convenient	0.698	(0.264, 1.848)	0.469
General	2.397	(0.824, 6.978)	0.109
Inconvenient	0.753	(0.339, 1.675)	0.487
Very inconvenient (reference)			
**The normality of doctors' treatment behavior**			0.121
Very normative	2.953	(0.832, 10.477)	0.094
Normative	2.658	(0.932, 7.580)	0.067
General	3.245	(1.339, 7.867)	0.009
Non-normative	2.464	(0.944, 6.434)	0.065
Very non-normative (reference)			
**The effectiveness of medical insurance in reducing the economic burden of disease**			
Effective	5.630	(2.861, 11.079)	<0.001
Not effective (reference)			

## Discussion

BMIURR can provide the basic medical security that rural residents need, promote satisfaction with the need for medical services, and effectively reduce the occurrence of poverty among rural residents due to illness. Therefore, it is crucial to improve the motivation of rural residents to ensure their willingness to participate in medical insurance. The results of the study indicate that there are four main factors affecting the willingness to participate: the familiarity with the medical insurance policies, the reasonability of medical insurance premiums, the normality of doctors' treatment behavior, and the effectiveness of medical insurance in reducing the economic burden of disease.

Rural residents with better recognition of the medical insurance policy were more likely to participate than those with poor recognition, which is consistent with the results of another study (15). The survey found that the number of rural residents who did have a certain understanding of the medical insurance system was larger than the number of those who did not. This may be due to the positive publicity achieved by rural officers and medical insurance agencies in raising medical insurance awareness. Furthermore, the KAP model predicts that people will adopt certain behaviors based on three stages: knowledge acquisition, attitude generation, and practice formation (16). Once rural residents can recognize the benefits that health insurance policies can bring to individuals, this may increase their willingness to participate in BMIURR.

The study found that the willingness to participate in BMIURR was higher among rural residents who considered the premiums for health insurance to be largely reasonable. Since the BMIURR was established, premiums have been gradually increasing to improve health insurance benefits. Although the annual income of rural residents has been increasing in recent years (17), their health service utilization equity has not improved as much as in urban areas (18, 19). As a result, they are not very willing to participate in health insurance. Some research has also found that the high fee for medical insurance is one reason why American farmers do not participate (20). For those with high incomes, health insurance premiums have no impact on their quality of life and are more motivated to purchase health insurance. On the other hand, low-income rural residents are dissuaded from the positive benefits of participating in medical insurance due to increasing premiums (21). Rural households have lower annual incomes, and as premiums increase, the proportion of medical expenses gradually increases, which may have an impact on normal household expenses, thus reducing the willingness to participate in insurance. I Especially if the individual is in good health, they are more reluctant to be insured.

In addition, the normality of doctors' treatment behavior was a significant variable in rural residents' willingness. The professional ethics of doctors in the appointed medical institution, the rational use of drugs, and the understanding of the medical insurance policy affected the satisfaction with the medical insurance. At present, the growth of medical expenses is mainly caused by non-normality medical service behavior, which is the increase in non-normality expenses caused by excessive medical treatments (22). Dranove suggested through a model that doctors tend to over-medicate, as evidenced by excessive test recommendations, leading to increased costs for tests (23). Although the current medical insurance payment method plays a role in controlling medical insurance fees, it has little effect on regulating the medical behavior of hospitals and doctors (24, 25). Nowadays, the drug proportion continues to decrease, but total medical expenses are growing rapidly. In the implementation of the “drug proportion” policy, the partial control of the drug proportion was found to induce excessive physical examinations, which meant that the policy did not fundamentally regulate medical behavior or medical expenses (26). The total payment control of basic medical insurance, which is currently widely practiced in China, is mainly aimed at controlling medical insurance expenses at medical institutions and indirectly regulating the medical behavior of doctors. However, in the implementation, it was found that the ability to guarantee the quality of medical services was weak, and when the quality of supervision was insufficient, the patient's timely use of essential medical resources was affected, especially patients with serious diseases. Patients may be reluctant to make enrolment because they do not receive access to basic healthcare resources during healthcare utilization (27). The Medicare physician system extends the supervision of the medical institution to the doctor by directly linking to the doctor's examination. The aim of the system is to regulate the medical service behavior of doctors (including diagnosis and prescription) to insure the reasonable use of Medicare funds. However, upon being implemented, the system was found to lack constraints in terms of policies and regulations, meaning that the cost of violations and warnings was minimal. Although it also includes many punitive measures, these can lead to a decrease in physician motivation. Meanwhile, Keenan et al. found that medical students similarly believed that physicians who violated program rules were less likely to be punished by official agencies and that the cost of punishment was lower (28), which could lead to a decreased likelihood that physicians will regulate their behavior.

With the new medical reform, the trend of the excessively rapid growth of medical expenses has been curbed and the proportion of individual health expenditures to total health expenditures has fallen to 28.8% in 2018 (29). The policy and associated measures play a role in regulating medical behavior, controlling medical expenses, and encouraging the sustainable development of medical insurance, but the normality of medical behavior still needs to be improved. However, most respondents who labeled the behavior of doctors as “non-normality” were found to be willing to participate in medical insurance, this may be related to the low payment ability of low-income rural residents and their greater dependency on medical insurance reimbursements. Additionally, the uncertainty of medical practice determines the uncertainty of the cost of care, as doctors have to choose different treatment options depending on the patient's condition. As a result, the cost of treatment for the same disease varies from patient to patient. The variability of diseases and the uncertainty of medical costs means that there will always be some benefits and some losses for the insurance provider, leading to the possibility of adverse selection by patients (30).

The results demonstrate that the effectiveness of medical insurance in reducing the economic burden of disease was highly related to willingness to participate. From a cost-benefit perspective, the public is likely to participate in insurance only when the benefits are greater than the costs. The economic burden of disease refers to economic loss due to illness, disability, or premature death of patients, and the economic loss of resources for the family and society tasked by caring for those affected by illnesses. Medical insurance plays an important role in reducing the financial burden of illness on the public, especially in terms of reduced personal health expenditures. Dou et al. (31) noted that the economic burden of disease of insured residents is lower than that of uninsured residents and that participation in medical insurance has a positive effect on reducing the economic burden of disease. Zhou et al. (32) also found that participation in health insurance had a positive effect on reducing the financial burden of illness. Having health insurance is a determinant of household catastrophic health expenditures for cancer patients in Korea (33), and having health insurance can effectively reduce household health expenditures. The incidence of catastrophic health expenditure for an insured family is significantly lower than that of an uninsured family (34).

However, the basic medical insurance level of coverage is still limited. When catastrophic health expenditure occurs, basic medical insurance will not be enough to relieve the heavy economic burden of disease. The World Health Organization argued that catastrophic health expenditures could create poverty if household health expenditures reach or exceed 50% of the household's non-food expenditure (35). There is a higher incidence of catastrophic health expenditures among households living in rural areas or poverty areas (36). The occurrence of severe diseases and their associated medical expenses can generate catastrophic health expenditures for uninsured households and there is therefore a positive relationship between serious diseases and poverty (37–39). The integration of urban and rural social health insurance has increased residents' utilization of health services but failed to reduce their financial burden of health care, especially for the poor (40). Therefore, although China initially established a relatively faultless basic medical system, it also needs to optimize the system design around the core function of “hedging financial risks” in medical security systems.

This study has several limitations. First, data were collected through a self-assessment questionnaire, and therefore, subjective bias may exist between the actual effect and the measured result. Second, due to the limitation of time and resources, only three provincial participants were selected as the subjects of this study, and the results may not be extended to other regions in China. Finally, the results cannot be interpreted as causality due to the cross-sectional design. To get more accurately assess the willingness of residents to participate in medical insurance, further studies are recommended to develop a more comprehensive and effective evaluation tool and a thorough analysis of the influencing factors on the willingness to participate.

## Conclusion

The study of rural residents' willingness to participate in BMIURR is of great significance for the sustainable development of the health insurance system. According to the results, most rural residents were willing to participate in basic medical insurance. The familiarity with the medical insurance policies, the reasonability of medical insurance premiums, the normality of doctors' treatment behavior, and the effectiveness of medical insurance in reducing the economic burden of disease were the factors that had a significant relationship with rural residents' willingness to participate. Enabling the dissemination of Medicare information requires regular professional training of the staff in the Medicare department and the appointed medical institution so they understand the content and changes in Medicare policy on a timely basis. To solve the problem of doctors' non-normality medical behavior, relevant departments should continuously improve related medical insurance management and take measures according to the present situation. Last but not least, the Chinese government should accelerate the establishment of the medical bottom-line guarantee mechanism for the poor.

## Data availability statement

The raw data supporting the conclusions of this article will be made available by the authors, without undue reservation.

## Ethics statement

The study protocol was reviewed and approved by the Research Ethics Committee of Harbin Medical University. Within the ethics and consent to participate declaration, this study adopted the form of verbal consent. Before conducting the investigation, the participants were explained the purpose of the questionnaire, and then the participants were asked for verbal consent. After the participants agreed, the questionnaire would be conducted. Because the survey participants are rural residents, most of them have limited educational levels. A questionnaire investigation was conducted with the participants' verbal consent.

## Author contributions

GT conceived the idea and wrote the manuscript. JX and TZ designed this study and conducted statistical analyses. ZK contributed to the writing-review and editing and funding acquisition. HZ, JL, QS, and FD acquired the data and contributed additional advice regarding the analysis. FC and JH provided constructive suggestions for the discussion of the manuscript. QW and HW critically revised the paper. All authors read and approved the final manuscript.

## Funding

The research was supported by National Natural Science Fund of China (72074064 and 71573068) and National Social Science Fund of China (19AZD013).

## Conflict of interest

The authors declare that the research was conducted in the absence of any commercial or financial relationships that could be construed as a potential conflict of interest.

## Publisher's note

All claims expressed in this article are solely those of the authors and do not necessarily represent those of their affiliated organizations, or those of the publisher, the editors and the reviewers. Any product that may be evaluated in this article, or claim that may be made by its manufacturer, is not guaranteed or endorsed by the publisher.

## Abbreviations

BMIURR: Basic medical insurance for urban and rural residents.
